# Drug-induced gingival overgrowth: FAERS based real-world pharmacovigilance study and network pharmacology analysis

**DOI:** 10.3389/fphar.2026.1775430

**Published:** 2026-04-02

**Authors:** Qingxia Zhu, Zebing Wu, Xianting Jiao, Lu Han, Yongfang Yuan

**Affiliations:** 1 Department of Pharmacy, Shanghai Ninth People’s Hospital, Shanghai Jiao Tong University School of Medicine, Shanghai, China; 2 College of Clinical Pharmacy, Shanghai Jiao Tong University School of Medicine, Shanghai, China; 3 Department of Pediatric infectious, Xinhua Hospital Affiliated to Shanghai Jiao Tong University School of Medicine, Shanghai, China

**Keywords:** bisphosphonates, drug-gene interaction, drug-induced gingival overgrowth, FAERS, pharmacovigilance

## Abstract

**Background:**

Drug-induced gingival overgrowth (DIGO) as an interdisciplinary clinical challenge, faces dual therapeutic barriers due to its unclear pathogenesis and high recurrence rate, forcing clinicians to balance therapeutic benefits against oral complications. Analyzing drug-adverse reaction links via real-world evidence can shift treatment from passive intervention to proactive prevention. This study aimed to identify DIGO-associated drugs using the US Food and Drug Association Adverse Event Reporting System (FAERS) data and to validate signals through pharmacology-guided multivariate logistic regression, thereby improving signal robustness and clinical interpretability.

**Methods:**

FAERS data (2004Q1–2024Q4) were analyzed. Multiple disproportionality methods including reporting odds ratios (RORs), proportional reporting ratios (PRRs), Bayesian confidence propagation neural network (BCPNN), and multi-item gamma Poisson shrinker (MGPS) were employed for signal detection, with Fisher’s exact test and false discovery rate (FDR) correction applied to control for multiple testing. Positive signals were subsequently validated through multivariate logistic regression and time-to-onset (TTO) analysis. Network pharmacology incorporating Kyoto Encyclopedia of Genes and Genomes (KEGG) and Gene Ontology (GO) enrichment analyses to delineate underlying molecular pathways.

**Results:**

Among 22,375,298 reports, 1,835 cases of gingival hyperplasia and 11,145 gingival lesions were identified. Bisphosphonates, alongside calcium-channel blockers (CCBs), immunosuppressants (IMMs), and antiepileptics (AEPs), showed significant associations (p < 0.0001). Zoledronic acid (ROR = 3.23, 95% CI: 2.24–4.67) and pamidronate (ROR = 5.33, 95% CI: 2.8–9.92) showed strong signals. TTO analysis indicated prolonged bisphosphonate use increased DIGO risk. Network analysis suggested possible involvement of Phosphatidylinositol 3-Kinase/A Kinase T (PI3K-AKT) and Mitogen-Activated Protein Kinase (MAPK).

**Conclusion:**

CCBs carried the highest DIGO risk, followed by IMMs and AEPs. Bisphosphonates were associated with DIGO and may act through PI3K-AKT/MAPK pathways, providing a mechanistic hypothesis for clinical medication safety.

## 1 Introduction

DIGO is a well-documented adverse effect associated with specific pharmacological agents. It is characterized primarily by excessive proliferation of fibrous tissue and significant enlargement of gingival volume (Droździk and Droździk, 2023). Gingival tissue serves as a crucial component of the natural immune defense system while simultaneously fulfilling essential functions in mastication, phonation, and facial esthetics. Pathological changes in gingival tissue can compromise quality of life significantly.

Clinical evidence identifies three principal categories of pharmacological agents associated with drug-induced gingival hyperplasia: calcium-channel blockers (CCBs; prevalence of 5%–20%), immunosuppressants (IMMs; 25%–30%), and antiepileptics (AEPs; 5%–50%). However, the precise pathophysiological mechanisms underlying this condition have yet to be elucidated (Droździk and Droździk, 2023; Chojnacka‐Purpurowicz et al., 2022; Brown and Arany, 2014; Trackman and Kantarci, 2015). Therapeutic approaches for drug-induced gingival hyperplasia encompass conservative and surgical modalities. Non-surgical management consists primarily of modification of the medication regimen (including dose adjustment or selection of an alternative drug) combined with conventional periodontal therapy. Surgical interventions, such as conventional gingivectomy or laser-assisted resection, are typically reserved for more severe cases (Campos et al., 2018). However, postoperative recurrence remains a significant clinical challenge, with reported rates varying depending on the causative medication and treatment modality employed (Lv et al., 2025; Hu et al., 2025).

In recent years, research has focused mainly on inflammation, the Wnt/β-catenin signaling pathway, epithelial–mesenchymal transition, and integrins (Ganesh, 2016; Larjava et al., 2014). Among them, the main clinical manifestations of gingival overgrowth caused by the calcineurin inhibitors cyclosporine and tacrolimus are inflammatory gingival hypergrowth and a small amount of fibrosis. The main clinical manifestations of gingival overgrowth caused by the CCBs nifedipine and amlodipine and sodium-channel blocker phenytoin are fibrosis. Fibrotic lesions of oral tissue can develop into oral cancer (Trackman and Kantarci, 2015; D’souza and Addepalli, 2018).

DIGO represents an uncommon adverse reaction to a drug. Conventional research approaches, including clinical trials, case reviews, and case reports, often encounter limitations such as insufficient sample sizes and relatively short study durations, which hamper epidemiological investigations. Pharmacovigilance studies have identified multiple drugs potentially associated with gingival hyperplasia (all belonging to the aforementioned three categories). However, a systematic and comprehensive analytical framework for establishing correlations between drug exposure and gingival hyperplasia has not been developed.

FAERS is a vital pharmacovigilance tool for detecting adverse drug events (ADEs). This post-marketing, spontaneous reporting system provides real-world ADE data for systematic analyses of drug safety. Hatahira et al. (2017) previously quantified the association strength between the aforementioned three drug classes and DIGO using this database, providing valuable references for clinical medication monitoring. However, the data only covers reports up to 2014, and due to accelerated drug innovation and upgraded adverse drug reaction reporting standards, the study suffers from issues of timeliness. At the molecular level, interactions between the drug and target protein generate therapeutic effects and ADEs. Network pharmacology can be employed to analyze networks of drug–gene interactions to elucidate drug-toxicity mechanisms, thereby providing insights into pathways of ADEs and supporting the safety of clinical medications.

We integrated the FAERS database and network-pharmacology methods to systematically explore the drug correlations and molecular mechanisms of drug-induced gingival overgrowth. We adopted a multidimensional analytical strategy. First, signal mining was conducted based on the FAERS database to identify drugs with positive signals. Then, a multiple-factor model was constructed to evaluate the correlation of drug exposure. Third, by integrating an open database of gene proteins, a network of drug–gene interactions was established and functional enrichment analyses conducted to clarify the potential molecular mechanism of action. Our study provides evidence-based information supported by multiple-source data for the safe use of clinical drugs.

## 2 Methods

### 2.1 Data source

This retrospective pharmacovigilance study used data from the FAERS database. This database provides information from patients or healthcare personnel using a spontaneous reporting system. We collected ADE data for 84 quarters from the first quarter of 2004 to the fourth quarter of 2024. FAERS data files contain seven datasets, including demographic and administrative information (DEMO), drug information (DRUG), adverse drug reaction information (REAC), patient outcome information (OUTC), information on report sources (RPSR), drug therapy starts dates and end dates (THER), and indications for use/diagnosis (INDI) (Chen et al., 2021).

### 2.2 Data mining

SAS 9.4 (https://www.sas.com/) was used for data analyses. Disproportionality analysis was employed as the primary data mining method for signal detection. This approach is the established regulatory-standard method for pharmacovigilance using spontaneous reporting databases such as FAERS. It is distinct from general-purpose data mining techniques (e.g., association rule mining, classification, or clustering), yet remains methodologically complementary to them. First, “gingival overgrowth” was targeted as the ADE. Cases with a reported level of “primary suspicion” were screened. The search results strictly followed the data-cleaning process recommended by the FDA, which included the removal of duplicate reports by selecting the PRIMARYID, CASEID, and FDA_DT fields from the DEMO table, sorting by CASEID, FDA_DT, and PRIMARYID in sequence. For reports with the same CASEID, the record with the latest FDA_DT was retained. Among reports with identical CASEID and FDA_DT, the one with the largest PRIMARYID was kept. Subsequently, the Preferred Terms (PTs) in all data were standardized and mapped to their corresponding System Organ Classes (SOCs) using the MedDRA 27.1 dictionary.

For signal detection, four disproportionality analysis methods were employed: ROR (Fusaroli et al., 2024), PRR (Fusaroli et al., 2024), BCPNN (Gosho et al., 2024; Huoshen et al., 2025) and MGPS (Heo and Jung, 2020). Supplementary Table S1 provides a comprehensive summary of each method, including the exact statistical metrics, signal detection criteria, parameter settings, and threshold values. In brief, a positive signal for ROR was defined as the number of reported cases ≥3 and the lower limit of the 95% confidence interval (CI) > 1; for PRR, a positive signal required the number of reported cases ≥3, PRR ≥2, and the corresponding χ^2^ ≥ 4 (Tada et al., 2020). These thresholds are consistent with MHRA standards. In addition, Fisher’s (Robertson et al., 2023; Benos et al., 2017) exact test was performed to calculate exact p-values for each drug-event combination, and the false discovery rate (FDR) correction was applied to control for multiple testing.

### 2.3 Time-to-onset (TTO) analysis

In the FAERS database, time-to-onset was defined as the interval between the start date of drug administration (i.e., START_DT) and the date when the adverse events first occurred (i.e., EVENT_DT). For the positive signals that were statistically significant in the multivariate logistic regression analysis (Bayman and Dexter, 2021), a further TTO analysis was conducted.

### 2.4 Drug–gene interaction (DGI) network and analyses

We used the search term “zoledronic acid” in the PubChem database (https://pubchem.ncbi.nlm.nih.gov/) to obtain the compound chemical structure and SMILES. We used the information in the CHEMBL database (www.ebi.ac.uk/chembl/) and STITCH database (http://stitch.embl.de/) to obtain the potential targets of the compound. The names of the obtained gene targets were standardized through the UniProt database (www.uniprot.org/). We employed the term “gingival overgrowth” in the GeneCards database (www.genecards.org/) and OMIM database (https://omim.org/) to obtain the potential targets of the disease.

The cross-potential target of zoledronic acid and gingival overgrowth was entered into the STRING database (https://cn.string-db.org/) and the species was restricted to *Homo sapiens*. The minimum required interaction score was set to medium confidence >0.4 for analyses. The obtained data for the protein–protein interaction (PPI) network was imported into Cytoscape 3.10.3 (https://cytoscape.org/) to calculate the degree of node connection (“degree”) and construct the PPI network. Using the “CytoHubba” plugin, 10 core targets were obtained.

For the biological functions of cross-potential targets, we conducted analyses of GO using the GO database and the “clusterProfiler” package of R 4.4.1 (R Institute for Statistical Computing, Vienna, Austria). This included enrichment analyses of the target genes for biological processes, cellular components, and molecular functions. Enrichment analyses were also conducted to determine the key pathways related to the metabolic effects of the target compounds on diseases using the KEGG database. Items with the species restricted to *Homo sapiens* and p < 0.05 were identified. Functional annotation using the GO database enabled selection of items with the top-15 p-values after correction by the Benjamini–Hochberg method. Analyses of signaling-pathway enrichment using the KEGG database permitted selection of items with the top 45 p-values after correction (Korthauer et al., 2019).

### 2.5 Statistical analyses

To further validate the signals identified by disproportionality analysis and to quantify adjusted risks, we adopted a twostep strategy: positivesignal drugs were first categorized by pharmacological class, and then multivariate logistic regression models were constructed within each class to estimate adjusted odds ratios while controlling for patient-level covariates. This approach enables better use of clinical information and reduces false-positive rates.

We focused on patients with gingival lesions (SMQ: 20000113) to identify risk factors for drug-induced gingival overgrowth in high-risk populations. Using R 4.4.1, we undertook logistic regression analyses (univariate and multivariate) to assess potential risk factors, including age, sex, bodyweight, geographic region of reporting, and primary suspected drug class. Model performance was evaluated using the C-index and corrected C-index, with values >0.9 indicating “excellent,” 0.7–0.9 “moderate,” and <0.7 “poor” predictive consistencies. Analyses of receiver operating characteristic curve (ROC) curves enabled measurement of specificity and sensitivity. Calibration curves and decision curves were used to validate the clinical utility of our model. Statistical significance was set at p < 0.05.

## 3 Results

### 3.1 Descriptive analyses

**FIGURE 1:**
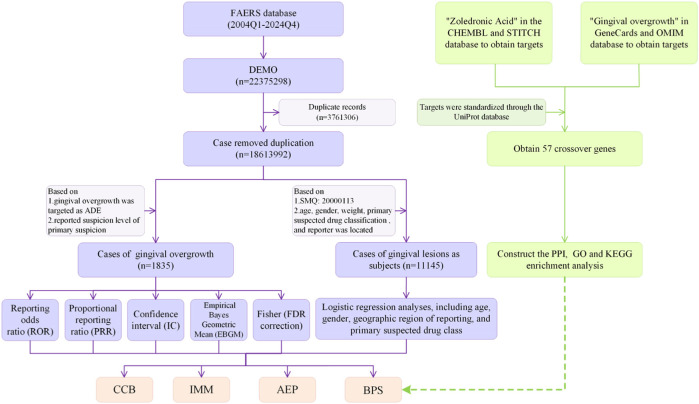
Data flow chart of FAERS data mining and network pharmacological analysis.

The initial dataset comprised 22,375,298 reported cases. Following duplicate removal, 1,835 cases of gingival overgrowth ADEs were retained for analyses (Figure 1). Demographic characteristics are presented in Table 1.

**TABLE 1:** Characteristics of cases associated with DIGO.

Characteristics	n (%)	Characteristics	n (%)
Sex	​	Geographic area of reporter	​
Female	833 (45.4)	Europe	663 (36.13)
Male	794 (43.27)	South America	47 (2.56)
Unknown	208 (11.33)	Asia	429 (23.38)
Age (years)	​	North America	551 (30.03)
<18	270 (14.71)	Oceania	19 (1.04)
18–44	306 (16.68)	Africa	17 (0.93)
45–65	512 (27.9)	Unknown	109 (5.93)
>65	277 (15.1)	Outcome	​
Unknown	470 (25.61)	Hospitalization	337 (18.37)
Median (years)	50	Congenital anomaly	9 (0.49)
Bodyweight (kg)	​	Required intervention	17 (0.93)
<80	283 (15.43)	Life-threatening	34 (1.85)
≥80	179 (9.75)	Death	43 (2.34)
Unknown	1373 (74.82)	Disability	88 (4.8)
Median	72.67	Other	1294 (70.52)
Reporter occupation	​	Unknown	305 (16.62)
Health professional	434 (23.65)	Classification	​
Physician	594 (32.37)	Serious	1530 (83.38)
Pharmacist	440 (23.98)	Non-serious	305 (16.62)
Consumer	251 (13.68)	​	​
Lawyer	8 (0.44)	​	​
Unknown	108 (5.88)	​	​

The cohort showed a slight female predominance (n = 833, 45.4%) compared with males (n = 794, 43.3%). The 45–65-year age group represented the largest proportion (n = 512, 27.9%), with a median age of 50 years. Most patients weighed <80 kg (n = 283, 15.4%), with a median bodyweight of 72.67 kg. Medical professionals (physicians (n = 594, 32.4%), pharmacists (n = 594, 32.4%), and healthcare workers (n = 434, 23.7%) submitted most reports. Geographically, European reporters predominated (n = 663, 36.1%). Notably, 83.4% (n = 1,530) of reports were classified as “serious,” with “other” outcomes being most prevalent (n = 1,294, 70.5%), followed by “hospitalization” (n = 337, 18.4%).

### 3.2 Signals of drug-induced gingival overgrowth

The positive signals related to gingival overgrowth were identified using the dual detection method of ROR and PRR with the threshold we set. The top-20 drugs in terms of frequency are detailed in Table 2.

**TABLE 2:** Drug-signal detection for DIGO drugs using ROR, PRR, IC, EBGM, and Fisher’s exact test from the FAERS database.

Drug name	N	ROR (95%CI)	PRR (χ^2^)	IC (IC025)	EBGM (EBGM05)	Fisher p (p fdr)	PI
Amlodipine	653	163.74 (148.80,180.18)	163.17 (67798.5)	6.72 (6.37)	105.46 (95.84)	0.00E+00 (0.00E+00)	Y
Cyclosporine	168	30.45 (25.98,35.68)	30.42 (4342.41)	4.79 (4.35)	27.73 (23.66)	1.62E-177 (1.24E-176)	Y
Phenytoin	138	83.29 (70.01,99.09)	83.08 (10349.6)	6.27 (5.38)	76.91 (64.65)	1.30E-205 (1.49E-204)	Y
Mycophenolic acid	64	9.90 (7.71,12.70)	9.90 (494.07)	3.26 (2.72)	9.59 (7.47)	2.63E-40 (1.21E-39)	Y
Tacrolimus	59	9.34 (7.21,12.11)	9.34 (425.25)	3.18 (2.62)	9.07 (7.00)	5.40E-36 (2.07E-35)	Y
Nifedipine	52	99.84 (75.75,131.60)	99.52 (4928.24)	6.60 (4.70)	96.73 (73.39)	3.17E-83 (1.82E-82)	Y
Valproic acid	49	10.38 (7.81,13.79)	10.38 (404.11)	3.34 (2.68)	10.13 (7.62)	3.02E-32 (9.91E-32)	N
Zoledronic acid	29	3.23 (2.24,4.67)	3.23 (44.04)	1.68 (1.04)	3.20 (2.22)	9.77E-08 (1.50E-07)	N
Levetiracetam	23	4.50 (2.98,6.79)	4.50 (61.80)	2.16 (1.37)	4.45 (2.95)	6.45E-09 (1.14E-08)	N
Diltiazem	23	19.01 (12.60,28.68)	18.99 (387.17)	4.23 (2.84)	18.77 (12.44)	1.13E-21 (3.24E-21)	Y
Carbamazepine	22	7.00 (4.60,10.66)	7.00 (111.73)	2.79 (1.86)	6.93 (4.55)	4.33E-12 (9.06E-12)	N
Ramipril	13	5.74 (3.33,9.90)	5.74 (50.50)	2.51 (1.32)	5.70 (3.31)	8.51E-07 (1.15E-06)	Y
Topiramate	12	4.37 (2.48,7.71)	4.37 (30.99)	2.12 (0.99)	4.35 (2.47)	3.18E-05 (3.85E-05)	Y
Pamidronic acid	10	5.33 (2.86,9.92)	5.33 (34.95)	2.41 (1.06)	5.30 (2.85)	2.81E-05 (3.60E-05)	Y
Lisinopril	10	3.75 (2.01,6.98)	3.75 (20.06)	1.90 (0.71)	3.74 (2.01)	4.64E-04 (5.08E-04)	Y
Amlodipine and atorvastatin	10	76.61 (41.12,142.74)	76.42 (740.29)	6.25 (2.41)	76.01 (40.79)	3.67E-16 (8.44E-16)	Y
Verapami	9	11.34 (5.89,21.83)	11.33 (84.39)	3.50 (1.56)	11.28 (5.86)	1.73E-07 (2.49E-07)	Y
Felodipine	9	116.34 (60.36,224.24)	115.90 (1020.21)	6.85 (2.30)	115.34 (59.84)	2.66E-16 (6.80E-16)	Y
Amlodipine and valsartan	6	8.04 (3.61,17.92)	8.04 (36.84)	3.00 (0.91)	8.01 (3.59)	1.29E-04 (1.48E-04)	Y
Rilonacept	5	93.65 (38.88,225.57)	93.36 (455.63)	6.54 (1.33)	93.11 (38.66)	3.52E-09 (6.74E-09)	Y

N, number of gingival hypertrophy; PI, package insert suggests risk for gingival hypertrophy; Y, yes; N, No.

Among the drugs suspected to cause ADEs in gingival overgrowth, amlodipine was reported most frequently (n = 653), followed by cyclosporine (n = 168) and phenytoin (n = 138). Pharmacological classification indicated that these drugs fell mainly into five major categories: CCBs (e.g., amlodipine), IMMs (e.g., cyclosporine), AEPs (e.g., phenytoin), bisphosphonates (e.g., zoledronic acid), and angiotensin-converting enzyme inhibitors (ACEIs; e.g., ramipril). We further performed TTO analysis for bisphosphonates that showed positive signals in both disproportionality analysis and multivariate logistic regression. The mean and median TTO for alendronate were 199.2 days and 163 days, respectively; for zoledronic acid, the mean was 208.1 days and the median was 91 days. Detailed TTO results are provided in Supplementary Table S3. In addition, cases of a combination of amlodipine and atorvastatin were observed. Drugs such as zoledronic acid, valproic acid, ramipril, and levetiracetam had positive signals, but the ADEs related to gingival overgrowth had not been included in their package inserts.

### 3.3 Analyses of risk factors

**FIGURE 2:**
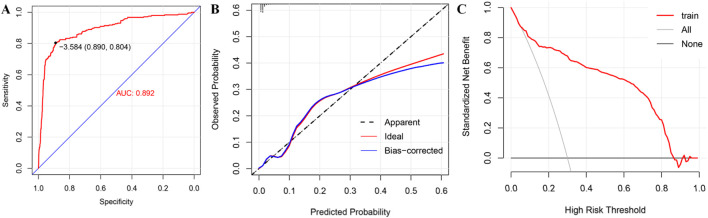
Parameter results of logist regression multivariate regression analysis. **(A)** ROC curve, **(B)** calibration curve and **(C)** decision curve of multi-factor model.

We used a logistic regression model to evaluate the risk differences of DIGO induced by different drugs. The area under the ROC curve (AUC) was 0.89 (95%CI = 0.87–0.92), the C index was 0.892, and the corrected C index was 0.887, indicating that the model had “good” discrimination. A sensitivity of 0.81 and specificity of 0.89 suggested “moderate” accuracy. The goodness-of-fit test (p = 0.092) confirmed that the model was “well calibrated” (Figure 2). Our study included 11,145 complete case data (excluding cases with missing risk factors), which are detailed in Supplementary Table S4, and the analytical variables included age, sex, bodyweight, classification of primary suspected drug (based on the criteria shown in Supplementary Table S5), and the region where the reporter was located.

**FIGURE 3:**
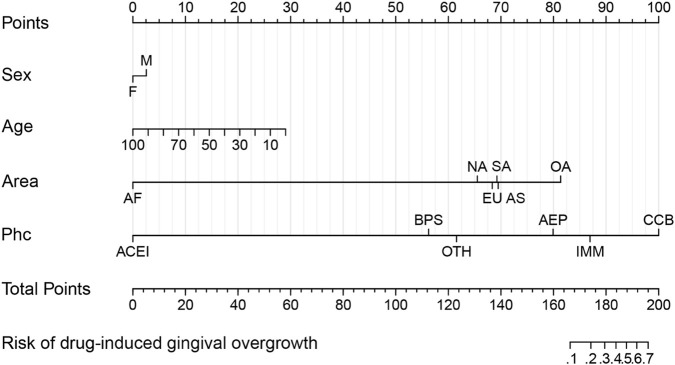
Nomogram of the risk factors for DIGO. Each risk factor was assigned a score on the scale axis. The total score was obtained by adding up the scores of each risk factor and projected onto the risk scale axis to estimate the probability of drug-induced gingival overgrowth. Abbreviations: AF, Africa; EU, Europe; AS, Subgroup; OA, Oceania; NA, North America; SA, South America; CCB, calcium-channel blocker; IMM, immunosuppressant; AEP, antiepileptic; OTH, other classes; BPS, bisphosphonate; Phc, pharmacological classification.

To control confounding factors, cases of single drugs were classified under the corresponding pharmacological classification, whereas cases of combined drugs were uniformly classified as “other.” After excluding non-significant variables (bodyweight, p = 0.07), through univariate analyses, the final model included four significant risk factors. To demonstrate their correlation more intuitively, a nomogram for predicting drug-induced gingival overgrowth was constructed based on the results (Figure 3). The risk score of male patients was significantly higher than that of female patients. Age increase was negatively correlated with risk. Regional differences were manifested as the highest risk among those reporting in Oceania. The classification of drug risk showed that CCBs, IMMs, and AEPs ranked among the top-three in terms of risk. The clinical risk score for ACEIs was significantly lower than that for bisphosphonates, though the risk of gingival overgrowth was not mentioned in the leaflet inserts of either type of drug.

### 3.4 DGI network

**FIGURE 4:**
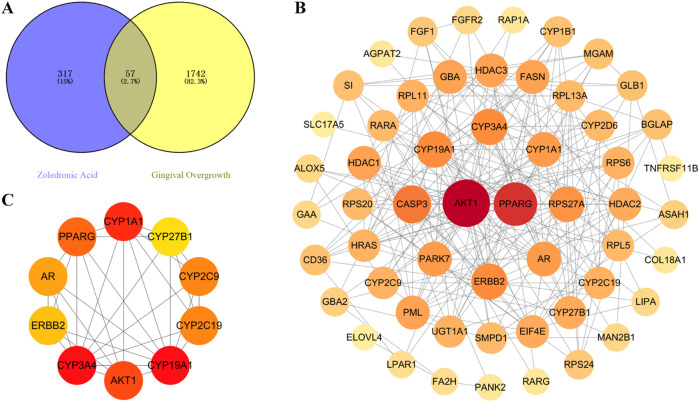
DGI network analyses. **(A)** Zoledronic acid - a potential target for gingival overgrowth Venn diagram, **(B)** Cross-potential PPI network of zoledronic acid and gingival overgrowth and **(C)** The top 10 genes with the highest degree value. Nodes represent proteins. Edges represent interactions. The larger the node area and the darker the color, the higher the degree value.

Risk-factor analyses showed that bisphosphonates were significantly associated with gingival overgrowth. To clarify the mechanism of action, we systematically analyzed the key targets and pathways of gingival overgrowth induced by bisphosphonates using network pharmacology. Taking zoledronic acid as an example, 57 cross-potential genes were screened out by integrating CHEMBL, STITCH, GeneCards, and OMIM databases (Figure 4A). After constructing a PPI network through the STRING database, an interaction network containing 55 nodes and 224 edges was obtained (Figure 4B), in which the node area and color depth reflected the degree value. Ten core targets were identified through the CytoHubba plugin: CYP19A1, CYP3A4, CYP1A1, AKT1, PPARG, CYP2C19, CYP2C9, AR, ERBB2, and CYP27B1 (Figure 4C). This approach provided a theoretical basis for the molecular mechanism of bisphosphonate-related gingival overgrowth.

**FIGURE 5:**
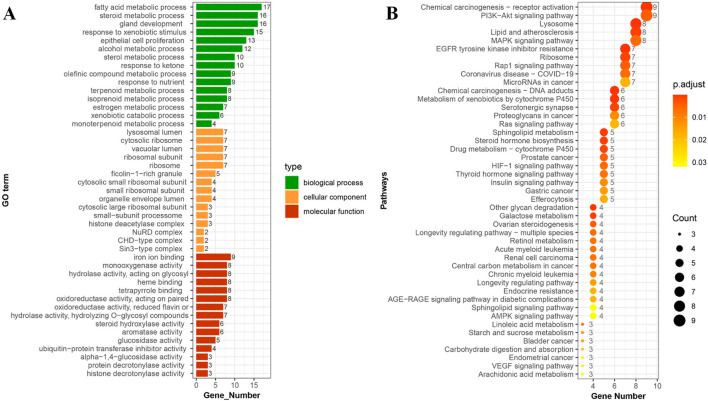
Integrated analysis of cross-potential genes between zoledronic acid and gingival overgrowth: GO functional enrichment analysis **(A)** and KEGG signaling pathway enrichment analysis **(B)**.

We undertook enrichment analyses of the cross-targets of zoledronic acid and gingival overgrowth using the GO database and KEGG database. Use of the former led to identification of 1,148 biological processes, 24 cellular components, and 217 molecular functional items (Figure 5A). Among them, the 15 items with the highest significance indicated that the biological processes were mainly enriched in “fatty acid metabolism”, “steroid metabolism”, and “epithelial cell proliferation.” Cellular components were concentrated in structures such as “lysosomes”, “ribosomes” and “vacuoles.” Molecular functions were significantly associated with functions such as “iron ion binding”, “monooxygenase activity”, and “glycosidic bond hydrolase activity”. Use of the KEGG database screened out 57 signaling pathways (Figure 5B). The top-45 significant pathways mainly involved key regulatory networks such as the activation of “chemocarcinogenic receptors”, the “PI3K-AKT signaling pathway”, and the “MAPK signaling pathway”. These data suggested that these pathways may be involved in the molecular regulatory mechanism of zoledronic acid-related gingival overgrowth.

## 4 Discussion

The present study involved the data of 1835 patients with drug-related gingival overgrowth. Demographic characteristics showed a balanced distribution between sexes (43.27% males and 45.4% females). However, multivariate analysis revealed that the scores of male patients were significantly higher than those of female patients (p < 0.05), which might have been related to the higher prevalence of tobacco smoking in men. Tobacco smoking is more likely to induce oral inflammation and increase the risk of gingival overgrowth. The age distribution showed a right-skewed trend, with the middle-aged and older group (>44 years) accounting for 43%. However, multivariate analyses indicated that the risk of disease onset decreased with age, which was speculated to be related to the higher use of CCB antihypertensive agents among older patients.

The source of ADE reports was mainly medical professionals (80%), and they covered three major regions—Europe (36.13%), North America (30.03%), and Asia (23.28%). Individuals represented diverse ethnic backgrounds, including Caucasian, East Asian, and African descent. We discovered that 83.38% of ADEs reached a “severe” level, among which 18.37% required hospitalization. These data suggested that drug-induced gingival overgrowth had a significant impact on the quality of life of patients, and that clinical intervention should be strengthened.

DIGO is a relatively rare ADE. Hence, the frequency-screening method was adopted to select the top-20 positive-signal drugs with the highest number of reported cases when analyzing monitoring data for ADEs. The research subjects covered three types of drugs known to be closely related to gingival overgrowth (CCBs, IMMs, and AEPs), as well as bisphosphonates (zoledronic acid and pamidronic acid) and ACEIs (ramipril and lisinopril). Among CCBs, amlodipine showed a disproportionately high number of ADE reports for gingival overgrowth *versus* IMMs/AEPs. This result may have been linked to the United Kingdom National Institute for Health and Care Excellence guidelines recommending CCBs as first-line antihypertensive agents for patients aged >55 years, which would have expanded the exposure base (National Clinical Guideline Centre UK, 2011). The onset time differed significantly across categories (CCBs: 262 days; IMMs: 71 days; AEPs: 37 days), which reflected temporal characteristics rather than disparities in direct risk (Hatahira et al., 2017). All three drug classes function by inhibiting cellular cation influx. Research has demonstrated that nifedipine (CCB) and phenytoin (AEP) induce gingival overgrowth through upregulation of expression of TGF-β and CCN2, as well as stimulating fibroblast proliferation and collagen deposition (Thompson et al., 2010; Kim et al., 2013).

The mechanism by which a high level of inflammation and low level of fibrosis in IMM-like cyclosporine-induced gingival overgrowth is not known. Some studies have found that cyclosporine inhibits the interleukin (IL)-2-mediated activation and proliferation of T cells, and interferes with the immune response, which may induce a low level of fibrosis in oral tissues (Chojnacka‐Purpurowicz et al., 2022; Janikowska et al., 2018). This difference in molecular mechanism may be the key factor leading to the varying risk of gingival overgrowth among different drugs. Notably, gingival-overgrowth warnings are omitted in labels for some AEPs (e.g., valproic acid, levetiracetam), necessitating timely updates based on post-marketing ADE data.

We quantified the risk factors of drug-induced gingival overgrowth through multiple-factor model analyses. In the category analysis of “primarily suspected drugs,” the risk of gingival overgrowth caused by CCBs, IMMs, or AEPs decreased in the sequence CCBs > IMMs > AEPs, and combination therapy (e.g., amlodipine + atorvastatin) was the main source of risk for other drugs. There was no significant risk of gingival overgrowth in ACEIs (p = 0.78). The instructions for bisphosphonate drugs (zoledronic acid, pamidronic acid) do not record the adverse reaction of gingival overgrowth, and clinical reports are rare (Wang et al., 2024). Bisphosphonate drugs treat osteoporosis mainly by inhibiting osteoclasts. Common ADEs include digestive-tract symptoms and bone pain, and serious adverse reactions are jawbone necrosis and atrial fibrillation (Jelin-Uhlig et al., 2024; Deardorff et al., 2022). Studies have shown that zoledronic acid inhibits the differentiation of gingival fibroblasts through the TGF-β1/Smad2/3 pathway, (Komatsu et al., 2016), and long-term use can inhibit the proliferation of oral epithelial cells and induce apoptosis (Srivichit et al., 2022; Di Vito et al., 2023). Experiments have shown that zoledronic acid can promote *Porphyromonas gingivalis* infection and recruit immune cells through cytokines to trigger a severe inflammatory response (Sun et al., 2023; Cekici et al., 2014).

We wished to understand more deeply the relationship between bisphosphonates and gingival overgrowth. We took zoledronic acid as an example and conducted network pharmacology of zoledronic acid and gingival overgrowth. According to CHEMBL and OMIM databases, the core gene targets of the effect of zoledronic acid on gingival overgrowth and the metabolic pathways corresponding to the cross-potential gene targets were identified, providing a reference for its mechanism of action. Studies have shown that zoledronic acid may induce drug-induced gingival overgrowth by regulating PI3K-AKT and MAPK signaling pathways. TGF-β1 can activate the Smad signaling pathway while also activating PI3K-AKT and MAPK pathways. Among them, AKT1, as a member of the serine-threonine protein kinase family, participates in the regulation of epithelial-cell proliferation, and its abnormal expression is associated with tissue overgrowth (Hamidi et al., 2017). The MAPK pathway participates in tissue fibrosis but also regulates the release of pro-inflammatory cytokines (Fengyang et al., 2012; Hu et al., 2015; Yeh et al., 2020). Mechanistic analyses have indicated that PI3K-AKT and MAPK pathways can affect inflammation-related cytokines by regulating NF-κB expression. The inhibitory effect of zoledronic acid on epithelial cells and fibroblasts may increase the susceptibility of gingival tissue to bacterial infection, and then activate NF-κB transcription through the immune response, which may be the main mechanism of drug-induced gingival overgrowth.

## 5 Conclusion

We conducted a systematic investigation into the influencing factors and mechanisms of drug-induced gingival overgrowth based on a self-reporting system of ADEs. Through analyses of many samples, the risk levels of three major types of drugs causing gingival overgrowth were identified for the first time. CCBs carried the highest risk, followed by IMMs and AEPs. A correlation between bisphosphonates and gingival overgrowth was also observed. Using network pharmacology, we found that bisphosphonates may be associated with a “low fibrosis–high inflammation” hyperplasia pattern in gingival tissue, potentially involving PI3K-AKT and MAPK signaling pathways. These findings provide a hypothesis-generating mechanistic basis for clinical medication safety and warrant further experimental validation.

## Data Availability

The original contributions presented in the study are included in the article/Supplementary Material, further inquiries can be directed to the corresponding authors.
