# Supplementary material for: Drug-induced gingival overgrowth: FAERS based real-world pharmacovigilance study and network pharmacology analysis
Source: Front Pharmacol. 2026 Apr 2;17:1775430. doi: 10.3389/fphar.2026.1775430 (PMC13083118; doi:10.3389/fphar.2026.1775430)
Supplement: Supplementary file 1 [file Supplementaryfile1.docx]

**Supplementary Material**

Table S1 Classification of pharmacological effects.

| **Metric** | **Formula & Bound** | **Criteria** | **Reference** |
| --- | --- | --- | --- |
| ROR | ROR = (a / b) / (c / d) Lower_CI_ROR = exp(log(ROR) - 1.96 * se_logROR) Upper_CI_ROR = exp(log(ROR) + 1.96 * se_logROR) | a ≥ 3, 95%CI lower ROR > 1 | [1] |
| PRR | PRR = (a / (a + b)) / (c / (c + d)) Lower_CI_PRR = exp(log(PRR) - 1.96 * se_logPRR) Upper_CI_PRR = exp(log(PRR) + 1.96 * se_logPRR) | a ≥ 3, 95%CI lower CI > 1 | [1] |
| BCPNN | IC = log2{a(a+b+c+d)/((a+b)(a+c))} E(IC)=log2{(a+r11)(N+α)(N+β)/(N+γ)(a+b+α1)(a+c+β1)} IC025(lower limit of IC) =E(IC)-2SD | IC025 > 0 | [2,3] |
| MGPS | EBGM = a(a+b+c+d)/((a+b)(a+c)) EBGM05 =exp(ln(EBGM)-1.96*sqrt(1/a+1/b+1/c+1/d) | EBGM05 > 2 | [4] |
| FISHER | p=(a+b)! (c+d)! (a+c)! (c+d)! / a! b! c! d! n!  FDR(i)=P(i)*m/I=min{FDR(i),FDR(i+1)} | P-value < 0.05 | [5,6] |
| Logistic regression analysis | Logit[P(periodontitis report = 1)] = β₀ + β₁ × Drug Exposure + β₂ × Sex + β₃ × Age + β₄ × Weight Group + β₅ × Reporter Type + β₆ × Country + β₇ × Report Year Group OR=exp( β)  Lower_CI_OR = exp(log(OR) - 1.96 * se_logOR) Upper_CI_OR = exp(log(OR) + 1.96 * se_logOR) | P-value < 0.05 | [7] |

(ROR, Reporting Odds Ratio; PRR, Proportional Reporting Ratio; BCPNN, Bayesian confidence propagation neural network; IC025,lower limit of IC; MGPS, Muti-item Gamma Poisson Shrinker; FISHER,Fisher's exact test.)

Table S2. Drug-Signal detection results of DIGO drugs using ROR, PRR, IC, EBGM, and Fisher’s exact test from the FAERS database.


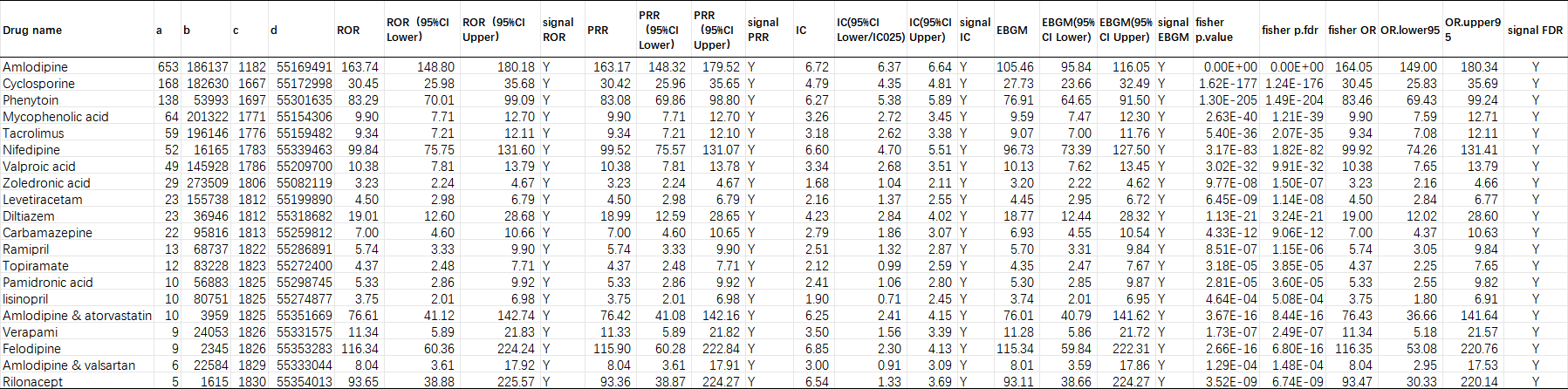


Table S3. The TTO result.

| **Drug name** | **Case** | **Mean** | **Median** | **Q1** | **Q3** |
| --- | --- | --- | --- | --- | --- |
| Alendronic Acid | 5 | 199.20 | 163 | 92 | 298 |
| zoledronic acid | 15 | 208.13 | 91 | 91 | 320.5 |

e.g.: Case, total number of reports related to DIGO for the drug; Mean, average time (in days) from drug initiation to DIGO onset; Median, the middle value of time to onset, with half of the reports before and half after; Q1 (first quartile), time when 25% of reports occur, representing the lower range; Q3 (third quartile), time when 75% of reports occur, representing the upper range.

Table S4 Multivariate logistic regression analysis of risk factors for drug-induced gingival overgrowth

| **Characteristics** | **Group level** | **No DIGO** | **DIGO** | **p.value** |
| --- | --- | --- | --- | --- |
| Gender | Total | 10783 | 362 | 5.528E-07 |
|  | Male | 3629(33.65%) | 171(47.24%) |  |
|  | Female | 7154(66.35%) | 191(52.76%) |  |
| Age | <18 | 274(2.54%) | 56(15.47%) | 3.794E-15 |
|  | 18-64 | 6189(57.4%) | 203(56.08%) |  |
|  | 65-85 | 4066(37.71%) | 103(28.45%) |  |
|  | >85 | 254(2.36%) | 0(0%) |  |
| Weight | <50 | 942(8.74%) | 58(16.02%) | 0.02713 |
|  | 50-100 | 8913(82.66%) | 283(78.18%) |  |
|  | >100 | 928(8.61%) | 21(5.8%) |  |
| Country | Africa | 18(0.17%) | 2(0.55%) | 2.2E-16 |
|  | Europe | 2174(20.16%) | 194(53.59%) |  |
|  | Asian | 1527(14.16%) | 48(13.26%) |  |
|  | Oceania | 42(0.39%) | 3(0.83%) |  |
|  | North America | 6648(61.65%) | 106(29.28%) |  |
|  | South America | 374(3.47%) | 9(2.49%) |  |

Table S5 Classification of pharmacological effects

| **Pharmacological classification** | **Drug name** |
| --- | --- |
| **Calcium Channel Blockers** | Nifedipine, amlodipine, lekadipine, nimodipine, Nicardipine, nigundipine, Nisodipine, felodipine, benidipine, Rasidipine, iratipine, diltiazem, verapamil, flunarizine, gylarizine, lidoflazine |
| **Antiepileptic drugs** | Phenytoin, carbamazepine, clobarazan, diazepam, gabapentin, levetiracetam, phenobarbital, pumidone, topiramate, valproic acid, zonisamide, lamotrigine, lacosamine, perampanide, oxcarbazepine, clonazepam |
| **Immunosuppressant** | Cyclosporine, tacrolimus, everolimus, sirolimus, mycophenolate mofetil, mycophenolate mofetil, prednisone, prednisolone, methylprednisolone |
| **Angiotensin Converting Enzyme Inhibitors** | Benazepril, Fosinopril, perindopril, Ramipril, midazepril, enalapril, captopril, quinazepril, lisinopril, qundopril |
| **Bisphosphonates** | Etedronic acid, parmiphosphate, alendronic acid, ibandronic acid, risedronic acid, zoledronic acid |

**Reference:**

[1] Fusaroli, M. et al., The Reporting of a Disproportionality Analysis for Drug Safety Signal Detection Using Individual Case Safety Reports in PharmacoVigilance (READUS-PV): Development and Statement. *Drug Safety* **2024,** *47* (6), 575-584.

[2] Gosho, M. et al., Multivariate generalized mixed-effects models for screening multiple adverse drug reactions in spontaneous reporting systems. *Frontiers in Pharmacology* **2024,** *15*.

[3]

[4] Heo, S. J. et al., Extended multi‐item gamma Poisson shrinker methods based on the zero‐inflated Poisson model for postmarket drug safety surveillance. *Statistics in Medicine* **2020,** *39* (30), 4636-4650.

[5] Robertson, D. S. et al., Online Multiple Hypothesis Testing. *Statistical Science* **2023,** *38* (4).

[6] Benos, P. V. et al., Fisher’s exact approach for post hoc analysis of a chi-squared test. *Plos One* **2017,** *12* (12).

[7] Bayman, E. O.; Dexter, F., Multicollinearity in Logistic Regression Models. *Anesthesia & Analgesia* **2021,** *133* (2), 362-365.
